# Eliminating *Aedes aegypti* from Its Southern Margin in Australia: Insights from Genomic Data and Simulation Modeling

**DOI:** 10.3390/insects17060623

**Published:** 2026-06-13

**Authors:** Gordana Rašić, Igor Filipović, Sean L. Wu, Tomás M. León, Jared B. Bennett, Héctor M. Sánchez Castellanos, John M. Marshall, Brendan J. Trewin

**Affiliations:** 1 Mosquito Genomics, QIMR Berghofer, Brisbane 4006, Australia; igor.filipovic@qimrb.edu.au; 2 Divisions of Epidemiology and Biostatistics, School of Public Health, University of California, Berkeley, CA 94720, USA; slwu89@berkeley.edu (S.L.W.); tomas.leon@cdph.ca.gov (T.M.L.); sanchez.hmsc@berkeley.edu (H.M.S.C.); john.marshall@berkeley.edu (J.M.M.); 3 Biophysics Graduate Group, Division of Biological Sciences, College of Letters and Science, University of California, Berkeley, CA 94720, USA; jared_bennett@berkeley.edu; 4 CSIRO Health and Biosecurity, Dutton Park, Brisbane 4102, Australia; brendan.trewin@csiro.au

**Keywords:** mosquito elimination, *Aedes aegypti*, population genomics, *Wolbachia*, incompatible insect technique (IIT), larval control, simulation modeling

## Abstract

A rare example of long-term elimination of *Aedes aegypti* occurred in Brisbane, where legislatively enforced removal of rainwater tanks led to its disappearance by the mid-1950s. However, widespread tank installation during a later drought reintroduced critical breeding habitat, enabling persistence in this subtropical region. With *Ae. aegypti* re-invading towns ~150 km north of Brisbane, we assessed the potential for sustained elimination. Using a genomics-informed simulation modeling framework, we integrated population genomic inference of connectivity and migration with entomological and environmental data to parameterize elimination scenarios. Genomic analyses revealed strong overall isolation among neighbouring populations in the region, with limited recent migration detected between the two southernmost populations, in Murgon and Wondai. We therefore focused simulations on these populations. Our results indicate that elimination is feasible with moderate releases of incompatible *Wolbachia*-infected (IIT) males (sorting error ≤ 10^−6^), provided non-compliant rainwater tanks are removed first. This combined strategy achieved >99% suppression and elimination in 35% of simulations in one town, whereas IIT alone resulted in moderate suppression (61–93%) and increased the risk of *Wolbachia* establishment at higher release ratios.

## Simple Summary

*Aedes aegypti* is the mosquito responsible for transmitting dengue, Zika, and several other viral diseases. After disappearing from southeast Queensland in the mid-1900s, this mosquito is now reappearing in towns closer to Brisbane, increasing concern about future disease risk. We combined mosquito genomic data with computer simulations to investigate whether these southern populations could be eliminated using currently available mosquito control tools. Our results suggest that these populations are genetically isolated and vulnerable to elimination, particularly when conventional source reduction is combined with releases of incompatible *Wolbachia*-infected male mosquitoes. This study highlights how genomic data and simulation modeling can help guide mosquito control strategies.

## 1. Introduction

The globally invasive mosquito *Aedes aegypti* is commonly known as the “yellow fever mosquito” [1], although in today’s world it is more important as the major vector of dengue, chikungunya, and Zika viruses. Population-genetic data, corroborated by historical records of yellow fever and dengue fever epidemics, indicate that *Ae. aegypti* left Africa some 500 years ago, first invading the Americas, and rapidly expanding across the Asia-Pacific region in the second half of the 19th century [1]. In Australia, the first reliable record of *Ae. aegypti*’s presence is a museum specimen collected in Brisbane in 1887 [2], but the first local dengue outbreaks occurred earlier and further north, in Townsville in 1879 and Charters Towers and Rockhampton in 1885 [3]. Although known as a vector of yellow fever at the time, *Ae. aegypti* was first implicated in the transmission of dengue fever by Thomas Bancroft, while investigating the Brisbane 1905 epidemic [4].

**Figure 1:**
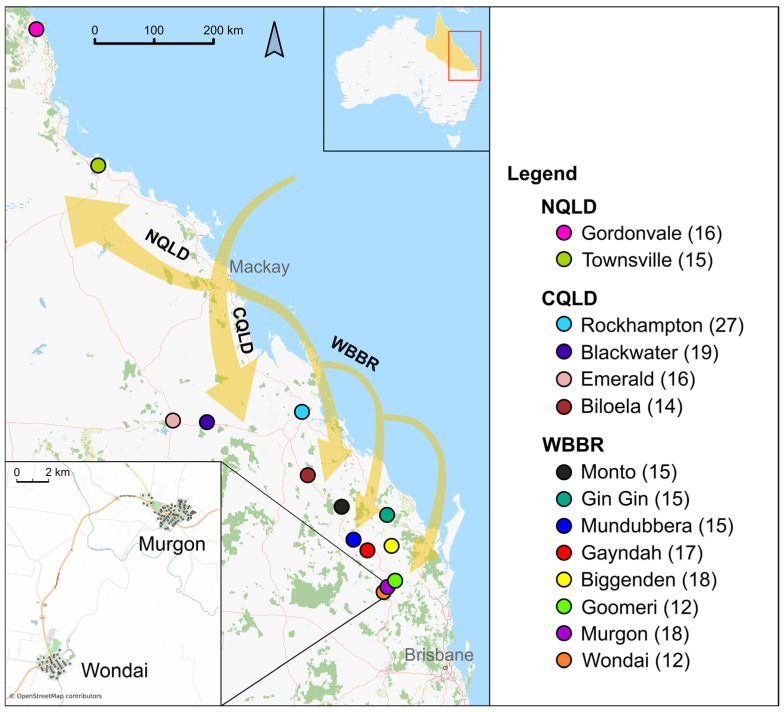
Sampling locations and the inferred population expansion of *Aedes aegypti* in Queensland. Color coding for towns is consistent throughout the manuscript figures. The number of genotyped mosquitoes in each town is shown in brackets. A map in the upper-right corner shows the current distribution of *Ae. aegypti* in Australia (yellow) and the sampled region (red rectangle). A map in the lower-left corner shows Murgon and Wondai with network nodes (centroids of residential blocks) used in the MGDrivE2 simulations. Yellow arrows depict genomically inferred population splitting and historical expansion throughout Queensland.

Until the late 1950s, this container-breeding mosquito was widely distributed throughout Australia’s north and west coast, and eastern seaboard for 3000 km north to south. It is hypothesised that intensive public health interventions after the Second World War led to a retraction of its populations into (sub)tropical central and north Queensland (Figure 1) [5]. These interventions implemented an approach that relied on intensive surveillance of residential areas to identify key larval mosquito production sources, and enforced compliance by insisting on removal of all water-holding rubbish and non-compliant rainwater tanks [6]. The removal or sealing of rainwater tanks is thought to have been a major factor in the elimination of *Ae. aegypti* from the southeast distribution margin, including the city of Brisbane, where they provided key habitat for continual larval development during the cold and dry winter months [6,7].

However, the presence of *Ae. aegypti* has been recently confirmed in the southern Wide Bay Burnett Region (WBBR), just 150 km north of Brisbane (Figure 1). Entomological surveys throughout WBBR since 2011 indicate that *Ae. aegypti* populations are larger and more widespread along major transport highways than previously thought (Supplementary Table S1). It is suspected that *Ae. aegypti*’s re-invasion of WBBR has been facilitated by region-wide installation of rainwater tanks and other changes in water storage practices in response to the region’s driest period on record, the “Millennium Drought”, between 2001 and 2009 [8].

While protected by *Wolbachia* in tropical north Queensland [9], communities in WBBR and central Queensland remain vulnerable to the spread of diseases vectored by *Ae. aegypti* (e.g., [10]). The 2019 dengue outbreak in Rockhampton, the first in over 60 years [11], typifies this vulnerability. The elimination of *Ae. aegypti* populations from the southern margin would not only benefit the towns involved but would also decrease the risk of southward re-incursion into the highly populated areas of southeast Queensland, including metropolitan Brisbane, where there are now over 300,000 rainwater tanks [7].

In theory, the removal of larval habitat would pressure vulnerable mosquito populations through extended dry and cold periods. O’Gower was the first to suggest that *Ae. aegypti* might be eliminated in areas of low rainfall by ‘a continuous mosquito control program, and complete replacement of rainwater tanks by a reticulated water system’ [12]. More recent mosquito control activities undertaken in WBBR’s town of Gin Gin (between 2014 and 2019) achieved suppression of *Ae. aegypti* numbers below detection threshold through the sealing of rainwater tanks, a targeted larvicide application to various larval breeding sites, and deployment of lethal adult traps [13]. This elimination approach requires extensive community engagement and access to private property for inspection, tank sealing, and chemical treatment of mosquito breeding sites by Government-licensed pest management technicians.

In parts of Innisfail in north Queensland, a strong suppression (but not elimination) of *Ae. aegypti* was achieved through releases of incompatible male mosquitoes over 20 weeks during the 2017–2018 field trial [14]. This biocontrol strategy, known as the Incompatible Insect Technique (IIT), is analogous to the traditional Sterile Insect Technique (SIT), but instead of releasing male insects sterilized by radiation, the released male mosquitoes carry the bacterium *Wolbachia* that causes death of embryos in matings with wild, or incompatible, females [15]. When released in numbers several times greater than the number of local males over the course of several months, incompatible males should effectively render all offspring of local females unviable and eventually crash the population [15]. Because IIT males can be released from moving vehicles along public roads (e.g., [16]), this strategy does not require access to private property unless required for entomological monitoring.

IIT could be a viable strategy for eliminating *Ae. aegypti* in WBBR, where most mosquito populations occur in small towns (with <1500 households) separated by an environment inhospitable to *Ae. aegypti*. Production of IIT males and their release across an entire small town, or even two neighbouring towns, should be operationally achievable in WBBR. If effective migration among towns is limited, elimination campaigns could be done successively across the region without much risk of quick mosquito re-invasion from untreated towns.

In this study, we use genomics-informed simulation modeling to assess if IIT and/or the removal of non-compliant rainwater tanks could lead to the local elimination of Australia’s southernmost *Ae. aegypti* populations. We employ a simulation modeling framework MGDrivE 2 [17], which enables simulations of seasonally-variable mosquito population dynamics and is capable of modeling different control strategies in spatially-structured mosquito populations [17]. To explore how vulnerable each WBBR town would be to mosquito re-invasion (post elimination), we ascertained historical and contemporary population connectivity through the analyses of population genomic data from *Ae. aegypti* collected across eight towns in WBBR and six towns located further north (Figure 1). The estimated recent migration rate, along with our entomological surveillance data and meteorological data, were used to calibrate the simulations of elimination campaigns in Murgon and Wondai, the two southernmost *Ae. aegypti* populations (Figure 1).

## 2. Materials and Methods

### 2.1. Mosquito Sampling

*Aedes aegypti* were surveyed and sampled across towns in the Wide Bay Burnett Region (WBBR) between February and April 2018, except in Gin Gin where the sample was collected in 2016 (the last year *Ae. aegypti* was detected in this town). Towns in central Queensland (CQLD) were surveyed and sampled from January to April 2019. The samples from north Queensland (NQLD) were described in two previous studies, as follows: *Ae. aegypti* from Gordonvale was collected in 2010 [18], and from Townsville in January 2014 [19]. Importantly, these NQLD samples were collected prior to the enrollment of the *Wolbachia* replacement program in the region [20,21]. In the WBRR, trapping was done across five premises, with one BGS trap and four ovitraps per premise, inspected weekly. Eggs were dried and stored in cooler bags, before being reared within until the fourth instar stage. In CQLD, adults were collected by the public health officials using Gravid Aedes Traps (GAT), and fourth instar larvae during house-to-house surveys. Adults and larvae were identified as to species [22], and *Ae. aegypti* were stored in 90% ethanol at −20 °C. To avoid the sampling of highly related individuals, we selected a single adult or larva from each trap at different collection dates where possible.

### 2.2. Mosquito Genotyping

Total genomic DNA was extracted from individual adults and larvae using DNeasy Blood and Tissue DNA extraction kit (Qiagen, Hilden, Germany), quantified with the Qubit High Sensitivity DNA kit (Thermo Fisher Scientific, Waltham, MA, USA), and 100 ng used for downstream processing. Double-digest Restriction site Associated DNA sequencing (ddRAD-seq, [23]) was used to generate genome-wide SNP and haplotype data for 229 *Ae. aegypti* individuals, following the previously described protocol for library preparation and sequence data processing [18]. We used the SAMtools genotype-calling algorithm in ANGSD [24].

### 2.3. Inference of Population Structure and History

To infer contemporary population genetic structure, we used the individual-based analysis implemented in RADpainter and fineRADStructure [25] with RAD haplotype loci generated in Stacks v.1.46 [26]. The input file for RADpainter was created using the script ‘Stacks2fineRAD.py’ that processes the output format of the Stacks populations program (‘batch_1.haplotypes.tsv’). First, the RADpainter program utilises the information from all SNPs within each RAD haplotype locus to find the most recent coalescence (common ancestry) among the sampled individuals. Second, the fineRADstructure program uses a MCMC scheme to cluster the individuals, retaining the population configuration with the highest probability. A visualization of the clustered coancestry matrix enables easy inference of the number of genetic clusters, quantification of ancestry sources in each group, and inference of relationships between groups through a simple tree-building algorithm.

Historical relationship among populations was inferred in Treemix [27] which produces a population tree under a model where contemporary populations are related to a common ancestor via a graph of ancestral populations. This tree is optimal for inferring the topology of relationships among populations, rather than the precise timing of demographic events. Using the frequencies of genome-wide SNPs from population samples, the pattern of population splits and mixtures is inferred through a Gaussian approximation to genetic drift. To infer the topology, we rooted the tree using a sample from La Lope, Gabon as an outgroup, given that this population resides in *Ae. aegypti*’s ancestral range [28]. We filtered 9954 SNPs that were found in the African and all Australian population samples, and used a window size of 50 (app. 2.5 Mb-blocks with 50 SNPs) to account for linkage between nearby SNPs. The analysis was repeated 1000 times to produce a consensus tree using the 90% support threshold.

Genetic clustering within WBBR was done using Discriminant Analysis of Principal Components (DAPC) [29], implemented in the R package adegenet v.1.42 [30]. DAPC finds linear combinations of alleles (also known as the discriminant functions, DFs) which best separate genetic clusters. We used the genetic clusters that were inferred in fineRADstructure, as well as a priori groups based on their sampling origin (towns). The R package plot3D was used to visualize the separation of clusters along the first three DFs.

To infer the direction of expansion into the WBBR, we first aimed to identify populations at or near the ancestral/source location, using the population-specific genetic parameter *β_st_* [31]. For a set of populations, those with the smallest *β_st_* should be the closest to the ancestral population (e.g., [32,33]). Negative *β_st_* is expected for large ancestral populations that have had time to accumulate many private alleles at low to intermediate frequencies. A linear increase in *β_st_* with the distance from the ancestral location is expected under the isolation-by-distance. Genome-wide *β_st_* and standard error were estimated using the R package FinePop2 v.0.2 [33], and the lm() function was used to estimate the slope of the linear relationship between *β_st_* and the Euclidean distance (in km) from the likely ancestral location.

### 2.4. Inference of Effective Population Size and Recent Migration

Contemporary effective population size (*N*_e_) was estimated for each WBBR population, using the linkage disequilibrium-based method as implemented in the program NeEstimator v.2.1 [34]. Each population had a different number of monomorphic loci and singleton alleles that were excluded from the total of 15,941 input loci, producing the estimates with 11,708 to 13,274 loci across eight populations.

We used the genetic assignment method in GenePlot [35] to identify recent migration events between WBBR populations. The method calculates the absolute fit of each individual to a given population, and the assignment results are presented graphically as log genotype probability (LGP) plots with graph positions for each individual. Every genotyped individual has some missing loci (commonplace for RADseq data), and GenePlot calculates appropriate plot positions for incomplete genotype profiles. Recent migrants are identified as individuals that do not have a good fit for the population where they were sampled from—i.e., have low LGP that falls outside the 1% quantile of the LGP distribution for the population of their sampling.

### 2.5. Simulations in MGDrivE 2

To model the expected performance of tank-sealing and IIT releases at suppressing and eliminating local *Ae. aegypti* populations in Murgon and Wondai, we used the MGDrivE 2 framework [17]. This framework models egg, larval, pupal, and adult mosquito life stages with overlapping generations, and larval mortality increasing with larval density. The inheritance pattern of *Wolbachia*, resulting from maternal transmission and cytoplasmic incompatibility, was modeled within the inheritance module of the framework. No fitness costs were assumed for *Wolbachia* carriers, which was a conservative estimate for modeling the risk of establishment during an IIT program. Because IIT programs require the release of males only, we incorporated imperfect sex sorting into the simulations by allowing a small proportion of *Wolbachia*-infected females to be released together with males. Female contamination rates ranged from 10^−6^ to 10^−9^, corresponding to approximately one infected female per million to one per billion released mosquitoes [16]. We distributed *Ae. aegypti* populations according to housing blocks, with a median of 17 houses per block, and block centroid coordinates calculated in QGIS 3.14.16. Each household was modeled with ~6 adult female *Ae. aegypti* during the peak season of year 1, based on the entomological surveillance data (File S1).

For each of the modeled elimination strategies, we ran 100 stochastic simulations for 5 years each in order to account for variation in the stochastic model and chance events such as population elimination. The modeled elimination strategies included the following:

- Scenario 1: Tank sealing (removal of non-compliant rainwater tanks) at the beginning of year 3,
- Scenario 2: Releases of IIT males at the beginning of year 4, twice a week for 12 weeks, with each release containing 14–40 times the number of males present in year 1 (the “overflooding ratio” of 14:1, 20:1, 30:1, 40:1), and
- Scenario 3: A combined strategy, with tank sealing at the beginning of year 3, and IIT releases at the beginning of year 4, twice a week for 12 weeks with an overflowing ratio of 14:1 (as compared to the number of males present during the peak season in year 1).

We assessed the effectiveness of each scenario by calculating (i) the proportion of simulations with zero adult females across blocks over the entirety of year 5, and (ii) the reduction in the total number of females per day, relative to simulations without any intervention, averaged across simulation runs.

Seasonal variation in *Ae. aegypti* population density was driven by local temperature and rainfall data, which determine adult mortality rates, juvenile development rates, and larval carrying capacity [17]. For the 5-year simulations, we used data for temperature and rainfall from the Australian Bureau of Meteorology for Murgon and Wondai for the years 2009 through 2013. The hourly temperature data were obtained from the weather station closest to Wondai (30 km south, Kingaroy Airport weather station; lat: 26.5737, lon: 151.8398). Daily rainfall data were obtained from the PostOffice station records in Murgon and Wondai. Temperature-dependent adult mortality and juvenile developmental rates were derived from the functions for *Ae. aegypti* from Mordecai et al. [36], and daily larval carrying capacity was derived using the method from White et al. [37] in which carrying capacity is proportional to past rainfall weighted by an exponential distribution with a mean of eight days. We calibrated static expected larval carrying capacities for each town based on non-time-varying simulations with fixed parameters, and then rescaled these values as rainfall-dependent time series using the static values as means. We further assumed that ⅙ of maximum larval carrying capacity in each town was provided by non-compliant rainwater tanks (permanent larval breeding sites), and the remainder by recent rainfall that fills up small breeding containers. This composition was based on the surveillance data from Goomeri to match their reduction in mosquito numbers observed one year after the rainwater tank-sealing campaign in 2018 (File S1).

Active mosquito movement between nodes was parameterized based on a mark–release–recapture experiment in Gin Gin [13], assuming that each adult mosquito has a 1% lifetime probability of movement to any neighbouring nide (housing blocks sharing a border), and with each potential destination node being assigned equal probabilities. The probabilities were converted into daily rates for the simulation model. Batch migration of immature and adult mosquitoes (passive migration) between Wondai and Murgon was parameterized using the genomics-inferred frequency of recent migrants (described below). MGDrivE 2 was implemented as a stochastic simulation model to capture random effects at low population sizes and the potential for population elimination. For each of the modeled elimination strategies, 100 stochastic simulations were run, and adult female mosquito counts (including those carrying *Wolbachia*) were recorded. Average mosquito life-cycle parameters used in these simulations are provided in Table S2.

### 2.6. Dispersal and Batch Migration Parametrization

We assumed each mosquito (both sexes) had a 1% lifetime probability to leave their natal node (block) and migrate to a neighbouring node (housing blocks sharing a border), and choice of specific destination node was given uniform probabilities. Because adult mosquito mortality is temperature dependent, we used the yearly average to calculate mean mosquito lifespan, from which we obtained the dispersal rate such that the probability of leaving the natal node before death was 1%.

We parameterized batch migration of egg and adult stages from Wondai to Murgon using the genetic assignment data. Genetic assignment revealed two individuals in Murgon for whom both parents had originated in Wondai, indicating these individuals were direct migrants, and an additional individual with mixed parentage (Wondai/Murgon) indicating it was the offspring of a direct migrant. Assuming the sample was unbiased, ~10% of the population in Murgon are direct migrants from Wondai. Using the estimate of 14,268 for the total mosquito population size in Murgon, the population who are not direct migrants is therefore 12,841. Of the remaining mosquitoes in the sample, we assume that they were offspring of unique mating pairs. To estimate the number of these parents which were from Wondai, assuming mosquitoes are randomly mating and that they were offspring of previous direct migrants (not direct migrants in the same sample), the number of parents from Wondai from sampled (without replacement) mosquitoes should follow a hypergeometric distribution. Using the maximum likelihood estimate for the parameters of the hypergeometric distribution, a final estimate of ~12.6% in Murgon are direct migrants. The rate parameter of the Poisson processes controlling the intensity at which batch migration occurred was fit such that on average that number of mosquitoes migrated from Wondai to Murgon each month.

## 3. Results

### 3.1. Genomic Analyses

We analysed 229 *Ae. aegypti* individuals collected from 14 localities, as follows: eight towns in WBBR (Monto, Gin Gin, Mundubbera, Biggendon, Gayndah, Goomeri, Murgon, Wondai), four towns in central Queensland, CQLD (Rockhampton, Blackwater, Emerald, Biloela), and two in north Queensland, NQLD (Gordonvale, Townsville) (Figure 1). The double-digest RAD sequencing with the previously validated protocol [18,19] generated a dataset containing 15,941 autosomal SNP loci with missingness up to 25% and a depth of ≥5×. Using the same filtering criteria in Stacks v.1.46, we obtained 7716 RAD haplotype loci (Supplementary Data S2).

#### 3.1.1. Genetic Structure and Demographic History of *Ae. Aegypti* in Australia Match Geography

**Figure 2:**
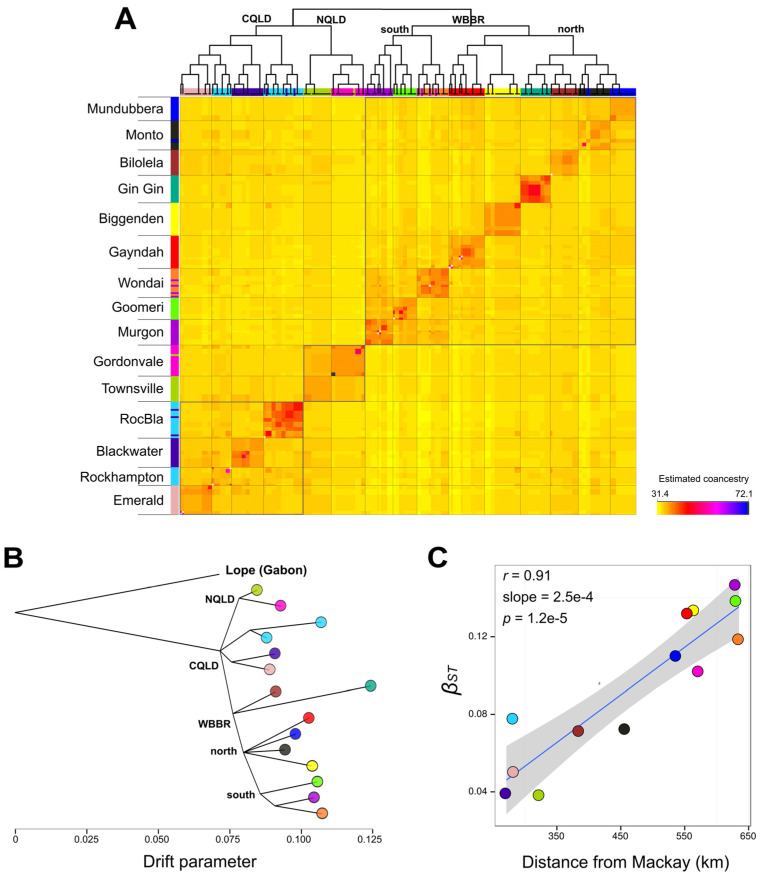
Contemporary genetic structure and historical relationship among *Aedes aegypti* populations in Queensland. (**A**) Clustered fineRADstructure matrix of coancestry averages per population (**B**) A rooted maximum likelihood tree of populations (consensus tree from 1000 Treemix analyses, all branches have ≥90% support). (**C**) Correlation between the population-specific differentiation and geographic distance from the hypothesised ancestral location (port of Mackay).

Contemporary genetic structure was ascertained using the individual-based analysis implemented in RADpainter and fineRADStructure [25]. The results show that *Ae. aegypti* from the same town share more coancestry with each other than with mosquitoes from different towns (Figure 2A). In other words, every town contains a genetically distinct population and these genetic groups (clusters) are visible as orange-red squares across the diagonal of the coancestry matrix (Figure 2A). There are some exceptions, as follows: a few individuals sampled in Murgon are grouped with individuals from neighbouring Wondai, and there was a cluster of individuals from Rockhampton and Blackwater, representing a family group. These cases indicate recent migrants.

The hierarchical structure among populations is clearly inferred (with all posterior probabilities > 0.95, Supplementary Figure S1), visualised as a tree at the top of the coancestry matrix (Figure 2A). Geographic region defines the highest hierarchical grouping, with a lineage of populations in central Queensland (CQLD), another lineage in north Queensland (NQLD), and the WBBR lineage that splits into the north-central group and the southern group of populations (Goomeri, Murgon, and Wondai). While falling under the jurisdiction of CQLD, Biloela is geographically close to the WBBR border and its *Ae. aegypti* population is more related to the WBBR lineage than to the CQLD lineage (Figure 2A).

To distinguish older and more recent splitting between identified populations, we used Treemix [27] that infers the topology of historical relationships among populations, under the assumption that their history is approximately tree-like. The resulting consensus population tree (Figure 2B) matches the hierarchical clustering inferred with fineRADstructure, as follows: the earliest population splitting corresponds to the three geographic regions—NQLD, CQLD, and WBBR. The population in Biloela, a town situated near the CQLD-WBBR border, is more related to the WBBR lineage than to the CQLD lineage. In WBBR, the pattern of ‘star-like’ branching in north-central populations (Monto, Mundubbera, Biggenden, Gayndah) indicates mass expansion from a common source. A stepping-stone southward expansion into Goomeri, Murgon, and Wondai formed another lineage (Figure 2B). The population in Gin Gin has undergone a substantial genetic drift, representing a genetic outlier in WBBR. This is was expected, as we sampled *Ae. aegypti* in this town only one year prior to the local elimination (i.e., at the very tail of the public health campaign).

#### 3.1.2. Serial North-to-South Expansion into WBBR Is Not from Rockhampton

To infer the direction of expansion from one ancestral source, we tested for correlation between the population-specific genetic parameter *β_st_* and geographic distance from the suspected ancestral location [32]. None of the analysed populations had a negative *β_st_*, indicating that we did not sample population(s) closest to the ancestral source, but the smallest value (*β_st_* = 0.04) was detected for Townsville (NQLD) and Blackwater (CQLD) (Figure 2C). Assuming the population origin on the coast between NQLD and CQLD, we considered Mackay, the first port of entry for European settlers on the Australian tropical coastlands [38], and found a highly significant positive correlation between *β_st_* and distance from Mackay (*r* = 0.91, *p* = 1.2 × 10^−5^, Figure 2C). The correlation was smaller when distance from Townsville was used (*r* = 0.77, *p* = 0.005) and non-significant if distance from Blackwater was used (*r* = 0.18, *p* = 0.563). The highest *β_st_* were recorded for populations at the most southern margin, 0.14 and 0.15, for Goomeri and Murgon, respectively. (Note that we excluded Gin Gin from this analysis as a demographic outlier with *β_st_* of 0.24).

#### 3.1.3. Most WBBR Populations Have Low Effective Population Size and High Isolation

**Figure 3:**
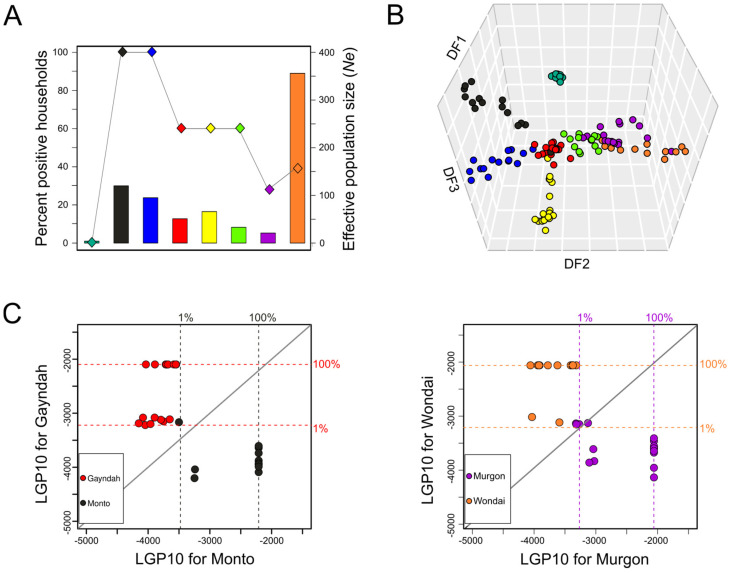
Effective population size and genetic isolation of *Aedes aegypti* populations in WBBR. (**A**) Effective population size (*N_e_*) and the percent of positive households for adult *Ae. aegypti* in eight towns in WBBR. (**B**) The first three axes from the DAPC with eight genetic groups in WBBR. (**C**) Genetic assignment analysis in GenePlot for detection of recent migrants between Monto and Gayndah (**left panel**), and Murgon and Wondai (**right panel**). Each individual is presented as a point that is color-coded according to the sampling location (e.g., black if sampled in Monto, red if sampled in Gayndah). 1% and 100% quantiles of the distribution of log genotype probabilities (LGPs) for each population are represented with dotted lines.

Excluding Gin Gin as a demographic outlier, genetic diversity of *Ae. aegypti* populations in WBBR decreased along the north-south direction, with significant correlation between latitude and the expected heterozygosity (*r* = 0.885, *p* = 0.008). The effective population size (*N_e_*) exhibited the same pattern, ranging between 21 and 120, but the most southern population in Wondai represented an outlier with *N_e_* that is 3–17 times greater than in other WBBR populations (Figure 3A). For *Ae. aegypti* collected in Gin Gin in 2016, *N_e_* was only 4 (95% CI: 3–4), clearly reflecting the effects of the public health campaign (2012–2016) that has led to undetectable mosquito populations from 2018 onwards [13]. The entomological survey in 2018 recorded 29–100% of inspected premises positive for adult *Ae. aegypti* (Figure 3A), and 20–100% positive ovitraps in WBBR towns (Table S1).

Genetic structuring in WBBR closely matches geography—the Discriminant Analysis of Principal components (DAPC) is able to group together individuals sampled in the same town (represented as points, color-coded based on the sampling location, Figure 3B). This multivariate analysis produced a clear separation of town samples (genetic clusters) across the first three axes, as follows: the first axis (DF1) separates Gin Gin from other populations, the second (DF2) separates the north-central from the southern populations, and the third (DF3) further separates populations within the north-central WBBR.

We used the genetic assignment visualisation method in GenePlot [35] to ascertain the individual’s absolute genetic fit to the inferred populations, and therefore to detect recent migrants as individuals with a good fit to populations other than where they were sampled. We found that one mosquito sampled in Monto showed a good fit to the Gayndah population (black point within red dotted lines, Figure 3C), indicating a putative first-generation migrant—i.e., an individual that was collected in Monto but has both parents from Gayndah. An individual that shows an equally good fit to both populations (its LGP falls on the thick diagonal line in the plot) represents a putative second-generation migrant, with one parent from each population. In Murgon, we detected one putative second-generation migrant and two putative first-generation migrants (Figure 3C), indicating a substantial recent gene flow from Wondai to the neighbouring Murgon. Given that female *Ae. aegypti*’s active flight range and intergenerational dispersal rarely exceed 200 m [39,40,41], the distance between towns in WBBR (at least 15 km) is certainly traversed passively through human transport such as commuting, cargo shipping, etc. Recent migrants were not detected in any other GenePlot analysis of population pairs, indicating high genetic isolation of most WBBR populations.

### 3.2. Simulation of Elimination Campaigns

With strong genomic evidence for southward expansion and contemporary migration between the two southernmost WBBR populations in Murgon and Wondai, we simulated the elimination programs that would simultaneously target both populations. Several assumptions used in the simulations were intentionally conservative, including the absence of *Wolbachia*-associated fitness costs and continued low-level migration between towns after suppression.

**Figure 4:**
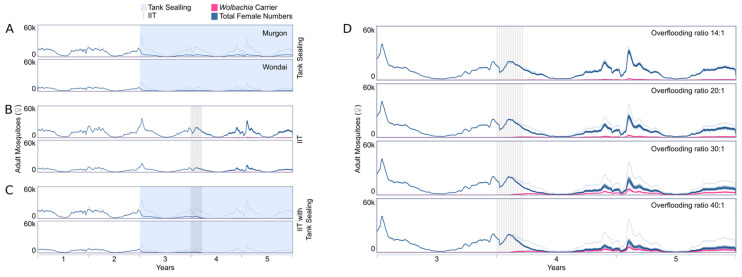
MGDrivE 2 simulations of tank sealing (source reduction), IIT (releases of *Wolbachia*-carrying males), and the combination of these two interventions in settings resembling Murgon and Wondai. Each setting is modeled as a metapopulation of residential blocks, and results represent the mean of 100 stochastic simulation runs. The resulting total adult female population size in Murgon and Wondai is denoted by black lines, and faint grey lines in the absence of interventions. (**A**) Scenario 1: tank sealing is modeled in Murgon and Wondai, starting at the beginning of year 3 and denoted by the shaded light blue portion of the plot. One sixth of the maximum larval carrying capacity in each town is assumed to be provided by non-compliant rainwater tanks that may be sealed. (**B**) Scenario 2: IIT releases are modeled in Murgon and Wondai, starting at the beginning of year 4 and occurring twice a week for 12 weeks, with each release (denoted by vertical black lines) containing 14 times the number of males as present in year 1 (the “overflooding ratio”). Due to imperfect sex sorting, *Wolbachia*-carrying females are included in the IIT releases at a frequency of 10^−6^. This leads to *Wolbachia*-carrying females emerging and persisting in each setting, denoted by magenta lines. (**C**) Scenario 3: tank sealing is combined with IIT releases at an overflooding ratio of 14:1. (**D**) Different overflooding ratios (14:1, 20:1, 30:1, and 40:1) for Scenario 2 in Murgon. Results suggest that a combined intervention of tank sealing with IIT releases could lead to local *Ae. aegypti* elimination for a moderate overflooding ratio of 14:1 (panel (**C**)). Neither intervention leads to elimination on its own (panels (**A**,**B**)), and larger IIT releases lead to establishment of *Wolbachia*-carrying females (panel (**D**)), hindering the impact of subsequent releases.

Seasonal fluctuation in both towns is very pronounced, with adult numbers peaking during summer (January–March) and reaching only 8.6% and 9.2.% of the peak size (in Wondai and Murgon, respectively) during the cold winter months (June–August). With the removal of non-compliant rainwater tanks (Scenario 1, Figure 4A), reduction in adult female numbers occurs quickly and persists through the following years, suppressing the population to 24% that of the non-treated population in each town. With 12 consecutive weeks of moderate IIT releases (Scenario 2, overflooding ratio of 14:1, Figure 4B), a small reduction in mosquito numbers was predicted the following year, as follows: 11.6% suppression in Murgon and 15.6% in Wondai (Figure 4B). A substantial population reduction (but not elimination) is only predicted with the highest overflooding ratio (40:1) and is comparable in impact to the tank-sealing campaign (~78% suppression, Figure 4D). Moreover, when the rate of contamination with *Wolbachia*-carrying females (due to imperfect sex sorting) is greater than 10^−7^, IIT with an overflooding ratio of 20:1 or more consistently leads to *Wolbachia* establishment (as shown by the solid magenta lines in Figure 4B,D).

Our simulations show that *Ae. aegypti* could be eliminated by implementing a combined strategy (Scenario 3), whereby a moderate IIT regime (overflooding ratio of 14:1) is conducted a year after the tank-sealing campaign (Figure 4C). In Wondai, complete elimination (zero females across all residential blocks over the entirety of year 5) was predicted in 35% of simulations, and in the remaining 65% of simulations local elimination was predicted in 95–98% of residential blocks. The total number of adult females per day in Wondai was on average 186 times higher in simulations without any intervention, which is equivalent to the average population suppression of 99.5%. Complete elimination was predicted in only 4% of simulations in Murgon, but local elimination was consistently predicted in >88% of residential blocks. The daily number of adult females in Murgon was suppressed by 99.2% (Figure 4C). For this combined strategy, *Wolbachia* establishment was not predicted even with the highest sex sorting error we simulated (10^−6^, Figure 4C).

## 4. Discussion

Mosquito elimination programs require a comprehensive effort supported by sustained funding, experienced staff, and community participation, as well as extended monitoring activities to confirm mosquito absence [42]. Very few successful attempts to eliminate established populations of *Ae. aegypti* through conventional programs have been reported to date. Such examples include the campaigns across South America between the 1940s and 1970s, and areas of Cuba in the 1980s that employed intensive insecticidal treatment and source reduction, but these programs were unsustainable [43]. The only long-term successful elimination of this disease vector appears to be in Brisbane, Queensland, where the legislatively enforced removal of rainwater tanks drove *Ae. aegypti*’s disappearance by the mid-1950s [6]. However, a decade-long drought during the 2000s led to the installation of hundreds of thousands of rainwater tanks throughout southeast Queensland, including Brisbane, providing potential critical larval habitat for *Ae. aegypti* persistence in this subtropical region [6]. With the discovery of *Ae. aegypti* populations in towns just 150 km north of Brisbane (Figure 1, south Wide Bay Burnett Region), we examined the potential for their elimination with accessible vector control tools.

Our entomological survey in the WBBR indicates that *Ae. aegypti* populations are larger than anticipated, but tend to decrease in southern towns (Figure 3A). Our genomic analyses support the hypothesis of *Ae. aegypti* serial north-to-south expansion into the WBBR (Figure 2B,C). Rockhampton was first hypothesised as a source for the linear expansion into WBBR by Kay et al. [5] based on intermittent *Ae. aegypti* surveys between 1956 and 1983. However, our genomic analyses indicate that the WBBR lineage and CQLD lineage (that includes Rockhampton) originate from the same ancestral region.

Most WBBR populations seem strongly genetically isolated (Figure 3B,C), which is favourable for achieving long-term elimination. While the number of mosquitoes sampled per town was modest, multiple independent analyses consistently recovered strong and biologically coherent population structure across the region. The high concordance between inferred demographic history, geographic distribution, and migration patterns suggests that stochastic effects associated with sample size did not obscure the major population genetic signatures relevant to this study. Importantly, the primary objective was to infer regional-scale connectivity and isolation patterns to inform elimination feasibility, rather than estimate fine-scale contemporary allele frequency dynamics.

A recent public health control program in the WBBR town of Gin Gin provides empirical evidence for our genomic-based inference. Namely, *Ae. aegypti* numbers in this town have remained below detectable levels three years after the completion of the public health mosquito control program [13]. Given the high level of genetic structuring in WBBR where each population has a specific genetic profile, ongoing monitoring should be able to distinguish between incomplete elimination and re-invasion of *Ae. aegypti* individuals from outside populations. Genomic data have been used to track *Aedes* invasions, including the recently established populations of *Ae. aegypti* in California [44]. Our study demonstrates remarkable power in ascertaining specific genetic profiles of invasive mosquito populations from neighbouring towns as close as 15 km (Figure 3B,C).

In addition to creating baseline population data for monitoring purposes, high-resolution genomics enabled us to quantify recent mosquito migration between Murgon and Wondai (genetic assignment test, Figure 3C). We used this genomically inferred migration rate to parametrize batch migration between towns in our spatially explicit simulations of elimination campaigns (MGDrivE2, Figure 4). To parameterize fine-scale dispersal rate (movement of *Ae. aegypti* between residential blocks within a town), we used the results of a mark–release–recapture (MRR) experiment in one of the WBBR towns [41]. However, recent advances in fine-scale spatial genomics, including analyses of separation distances between close kin, highlight this approach as a powerful alternative to MRR [39,45]. High-resolution genomics is expected to increasingly inform mosquito control modeling, surveillance, and decision-making [46].

Importantly, the objective of this study was not to define fixed operational parameters for an immediate intervention campaign, but rather to demonstrate how population genomic inference can be integrated with simulation modeling to assess elimination feasibility and guide strategic planning for mosquito populations in the region. Although the genomic samples analysed from WBBR were collected between 2016 and 2019, multiple analytical approaches consistently revealed strong population structuring and limited recent migration, indicating substantial regional isolation. Any future elimination program would nevertheless require contemporary entomological and genomic surveillance to refine model parameterization and account for temporal changes in mosquito abundance, connectivity, and environmental conditions.

Simulation modeling of current and historical *Ae. aegypti* persistence across Australia predicts high natural vulnerability of subtropical populations (such as those in WBBR) due to suboptimal climate [47]. However, this vulnerability depends strongly on the availability of large larval breeding sites like rainwater tanks [7,42]. The removal/sealing of non-compliant rainwater tanks should therefore significantly increase the probability of *Ae. aegypti* elimination in WBBR. The operational implementation of tank-sealing campaigns nevertheless presents substantial logistical and social challenges. Sustained source-reduction programs require community participation, access to private properties, ongoing compliance monitoring, and, in some regions, financial support mechanisms to facilitate modification of non-compliant infrastructure. These activities are labour-intensive and may be difficult to sustain over long periods without strong local government engagement. Nevertheless, historical elimination *of Ae. aegypti* from Brisbane [6] and recent suppression campaigns in WBBR towns ([13], File S1) demonstrate that targeted source reduction can be implemented by coordinated public health policy and community participation.

While sealing of non-compliant tanks seems insufficient to achieve elimination of *Ae. aegypti* on its own, our simulations show that its implementation ahead of the IIT campaign is critical (Figure 4). Namely, our simulated releases of *Wolbachia*-infected (IIT) males led to a moderate population suppression (~78% reduction in female *Ae. aegypti* numbers) even with a 40:1 overflooding ratio of IIT males to wild-type males (Figure 4B). However, when tank sealing was simulated one year prior to the IIT releases, highly effective suppression (>99% reduction in adult female *Ae. aegypti* numbers) was observed in both towns, and the complete elimination was observed in 35% of simulations in one town (Figure 4C)

An IIT field trial in Innisfail, northern Queensland achieved high suppression (>80%) across three treatment sites, with releases conducted three times a week over 20 weeks and an overflooding ratio of ~10:1 [14]. Our simulations of the releases conducted twice a week over 12 weeks predict that increasing the overflowing ratio from 14:1 up to 40:1 achieves suppression levels 61–93%, but larger releases (≥20:1) dramatically increase the chance of *Wolbachia* establishment if the sex-sorting error is greater than 10^−7^ (Figure S2). This is consistent with a recent modeling study of the IIT campaign in Innisfail [48], which found that a sex-sorting error rate of 10^−7^ or less is needed to achieve a low probability of *Wolbachia* establishment, along with ‘adaptive releases’ three times a week that start with an overflooding ratio of 5:1 or 15:1, and decrease in size as the population is suppressed over time [48].

Depending on the underlying technology, the reported sex-sorting error in IIT programs is between 10^−3^ and 10^−9^ [16,49], and we opted to simulate the releases with the sorting error rates from 10^−6^ to 10^−9^ (File S1). It is worth noting that our simulations could be overestimating the risk of *Wolbachia* establishment under imperfect sex sorting, as they assumed that *Wolbachia* infection had no fitness cost. While the laboratory experiments revealed that transfections with *Wolbachia* strains like *w*AlbB and *w*Mel had no significant cost for *Ae. aegypti* males [50,51], *Wolbachia* decreased female fecundity and survival, as well as hatching of eggs that were quiescent in warm environments [52]. However, field data from recent IIT trials in Singapore reported *Wolbachia* (*w*AlbB) establishment in some treatment areas with an overflooding ratio of 30:1, even with the deployment of a sex-sorting technology with the highest fidelity [49]. One mitigation strategy for this issue is the irradiation of IIT mosquitoes (SIT-IIT) to ensure that accidentally released *Wolbachia*-carrying females are also sterile [49]. Another approach to minimize the chance of *Wolbachia* establishment could be to use a *Wolbachia* strain with high fitness cost like *w*MelPop [53]. Such a strain is difficult to establish even with very high numbers of released *Wolbachia*-carrying females, and if established, would likely cause a population crash during dry winter months in WBBR because it causes high egg mortality under such conditions [54,55]. The release ratio and frequency for the *w*MelPop-carrying males, like for the irradiated IIT males, would have to be high. Conversely, our simulations indicate that *Ae. aegypti* populations in WBBR could be eliminated with a moderate release frequency and numbers of IIT males sorted under moderate error rate (10^−6^), if non-compliant rainwater tanks are removed first (Figure 4C).

Recent large-scale field deployments of *Wolbachia*-based incompatible insect technique (IIT) in urban settings have demonstrated substantial suppression of *Aedes aegypti* and *Ae. albopictus* populations (often exceeding 70–80%) and associated reductions in dengue incidence [56,57,58]. However, these outcomes have required sustained releases over extended periods, and complete elimination has not been consistently achieved [49,56,57,58]. Our results are consistent with these findings and extend them by demonstrating that integrating larval source reduction with IIT can substantially increase the probability of elimination, particularly in genetically isolated populations at range margins. Importantly, any large-scale biocontrol strategy, including *Wolbachia*-based IIT programs, requires sustained monitoring of ecological and epidemiological outcomes over time. Recent deployment examples highlight post-release surveillance and adaptive management in a continuous highly urbanized context [49]. Developing scalable surveillance approaches for geographically broad and fragmented regions, such as WBBR, remains an important consideration for long-term IIT implementation.

While many ecological and epidemiological considerations surrounding *Wolbachia* deployments are particularly relevant to long-term population replacement strategies, suppression-based IIT programs also require continued monitoring and adaptive management. Recent large-scale deployments in Singapore demonstrated substantial suppression of *Ae. aegypti* populations and associated reductions in dengue incidence, while also highlighting the importance of minimizing unintended *Wolbachia* establishment, maintaining release quality, and accounting for ecological and operational complexities in highly urbanized environments. Continued surveillance therefore remains important for evaluating long-term intervention performance and ecological stability under different environmental and operational conditions.

The importance of simulation modeling to predict the impact of integrated mosquito control programs tailored to specific ecological settings has been widely recognized in malaria elimination campaigns [59]. To our knowledge, this is the first modeling analysis of an integrated elimination strategy, combining source reduction and IIT against the dengue vector, *Ae. aegypti*. We considered the scenarios where rainwater tank sealing and the IIT campaign both start at the peak season of mosquito productivity. However, in regions like WBBR where mosquito populations experience highly seasonal dynamics, starting both interventions ahead of the peak season is expected to improve the effectiveness of a campaign. Finding solutions that maximise population suppression could be further tackled through the use of optimisation models for vector control [59]. Our estimates of intervention outcomes are conservative for informing a public health plan for the elimination of *Ae. aegypti* in the WBBR.

Future climatic and environmental changes may alter mosquito dispersal, persistence, and recolonization dynamics, particularly at the margins of the current distribution. Environmental conditions may also influence the long-term performance of *Wolbachia*-based interventions, including through effects on *Wolbachia* stability and mosquito population dynamics [60]. Incorporating future climate scenarios into genomics-informed simulation frameworks therefore represents an important direction for future work.

Here we suggest the benefits of combining larval source reduction with a novel technology like IIT to eliminate recently established *Ae. aegypti* populations at the range margin in Australia. These benefits are also likely to exist for similar populations in other parts of the world. For example, in less than a decade since its first detection in California [61], *Ae. aegypti* has established large populations that have proven difficult to suppress, as evidenced by the recent IIT trials in Fresno County [16]. During the same period, the state has been experiencing major droughts [62], which led to changes in water harvesting and storage policy, such as the introduction of the Rainwater Capture Act of 2012 [63], incentivising the installation of rainwater tanks on residential properties. Incorporating the sealing/removal of non-compliant water tanks into the mosquito control programs that utilise IIT could prove critical for the elimination of *Ae. aegypti* populations in California as well.

## Data Availability

Variant Call Format (VCF) file with genotypes across >15,000 autosomal loci for 229 *Aedes aegypti* analyzed in this study is available in supplemental materials. (Data_file_s1.vcf). Tab-delimited table (Data_file_s2.txt) showing centroid coordinates for each residential block, the number of households per block, and the number of human hosts per block in Murgon and Wondai. These data that were used to calibrate the spatially explicit simulations in MGDrivE 2 are available in Supplemental Materials. Raw demultiplexed sequencing data (fastq format) and associated metadata are publicly available at NCBI SRA under PRJNA75871, PRJNA241150, and PRJNA348645.

## Supplementary Materials

The following supporting information can be downloaded at: https://www.mdpi.com/article/10.3390/insects17060623/s1, Figure S1. Mosquito numbers in Wondai, WBBR; Figure S2. IIT releases with different sex sorting error rates. Figure S3. Mosquito numbers before and after the tank-sealing campaign in Goomeri, WBBR. Table S1. Results from the surveillance activities in the Wide Bay Burnett region (WBBR); Table S2. Average mosquito life cycle parameters. Data S1. Variant Call Format (VCF) file (Data_file_s1.vcf) with genotypes across >15,000 autosomal loci for 229 *Aedes aegypti* analyzed in this study. Data S2. Tab-delimited table (Data_file_s2.txt) showing centroid coordinates for each residential block, the number of households per block and the number of human hosts per block in Murgon and Wondai. These data were used to calibrate the spatially-explicit simulations in MGDrivE 2. References [64,65,66,67,68] are cited in the supplementary materials.

## References

[1] Powell J.R., Gloria-Soria A., Kotsakiozi P. (2018). Recent History of *Aedes aegypti*: Vector Genomics and Epidemiology Records. BioScience.

[2] Debenham M.L. (1987). Nomenclature, Synonymy, Literature, Distribution, Biology and Relation to Disease: Genus Aedes, Subgenera Scutomyia, Stegomyia, Verrallina. The Culicidae of the Australasian Region.

[3] McBride W.J. (2010). Dengue fever: Is it endemic in Australia?. Intern. Med. J..

[4] Bancroft T.L. (1906). On the aetiology of dengue fever. Australas. Med. Gaz..

[5] Kay B.H., Marks E.N., Barker-Hudson P. Dengue in Queensland, Australia, 1981–1983. Proceedings of the International Conference on Dengue and Dengue Haemorrhagic Fever.

[6] Trewin B.J., Darbro J.M., Jansen C.C., Schellhorn N.A., Zalucki M.P., Hurst T.P., Devine G.J. (2017). The elimination of the dengue vector, *Aedes aegypti*, from Brisbane, Australia: The role of surveillance, larval habitat removal and policy. PLoS Negl. Trop. Dis..

[7] Trewin B.J., Darbro J.M., Zalucki M.P., Jansen C.C., Schellhorn N.A., Devine G.J. (2019). Life on the margin: Rainwater tanks facilitate overwintering of the dengue vector, *Aedes aegypti*, in a sub-tropical climate. PLoS ONE.

[8] van Dijk A.I.J.M., Beck H.E., Crosbie R.S., de Jeu R.A.M., Liu Y.Y., Podger G.M., Timbal B., Viney N.R. (2013). The Millennium Drought in southeast Australia (2001–2009): Natural and human causes and implications for water resources, ecosystems, economy, and society. Water Resour. Res..

[9] Ryan P.A., Turley A.P., Wilson G., Hurst T.P., Retzki K., Brown-Kenyon J., Hodgson L., Kenny N., Cook H., Montgomery B.L. (2020). Establishment of *w*Mel *Wolbachia* in *Aedes aegypti* mosquitoes and reduction of local dengue transmission in Cairns and surrounding locations in northern Queensland, Australia. Gates Open Res..

[10] Hugo L.E., Stassen L., La J., Gosden E., Ekwudu O., Winterford C., Viennet E., Faddy H.M., Devine G.J., Frentiu F.D. (2019). Vector competence of Australian *Aedes aegypti* and *Aedes albopictus* for an epidemic strain of Zika virus. PLoS Negl. Trop. Dis..

[11] Walker J., Pyke A., Florian P., Moore F., Smoll N., Adegbija O., Khan A., Hasan R., Carroll H., Harris R.R. (2018). Re-emergence of dengue virus in regional Queensland: 2019 dengue virus outbreak in Rockhampton, Central Queensland, Australia. Commun. Dis. Intell..

[12] O’Gower A.K. (1956). Control measures for *Aedes aegypti*: Surveys in northern Australia. Health.

[13] Trewin B.J., Montgomery B.L., Hurst T.P., Gilmore J.S., Endersby-Harshman N.M., Crisp G.J. (2022). Extensive public health initiatives drive the elimination of *Aedes aegypti* (Diptera, Culicidae) from a town in regional Queensland: A case study from Gin Gin, Australia. PLoS Negl. Trop. Dis..

[14] Beebe N.W., Pagendam D., Trewin B.J., Boomer A., Bradford M., Ford A., Liddington C., Bondarenco A., De Barro P.J., Gilchrist J. (2021). Releasing incompatible males drives strong suppression across populations of wild and *Wolbachia*-carrying *Aedes aegypti* in Australia. Proc. Natl. Acad. Sci. USA.

[15] Laven H. (1967). Eradication of *Culex pipiens fatigans* through cytoplasmic incompatibility. Nature.

[16] Crawford J.E., Clarke D.W., Criswell V., Desnoyer M., Cornel D., Deegan B., Gong K., Hopkins K.C., Howell P., Hyde J.S. (2020). Efficient production of male *Wolbachia*-infected *Aedes aegypti* mosquitoes enables large-scale suppression of wild populations. Nat. Biotechnol..

[17] Wu S.L., Bennett J.B., Sánchez C.H.M., Dolgert A.J., León T.M., Marshall J.M. (2021). MGDrivE 2: A simulation framework for gene drive systems incorporating seasonality and epidemiological dynamics. PLoS Comput. Biol..

[18] Rašić G., Filipović I., Weeks A.R., Hoffmann A.A. (2014). Genome-wide SNPs lead to strong signals of geographic structure and relatedness patterns in the major arbovirus vector, *Aedes aegypti*. BMC Genom..

[19] Rašić G., Filipović I., Callahan A.G., Stanford D., Chan A., Lam-Phua S.G., Tan C.H., Hoffmann A.A. (2016). The queenslandensis and the type form of the Dengue Fever Mosquito (*Aedes aegypti* L.) Are Genomically Indistinguishable. PLoS Negl. Trop. Dis..

[20] Hoffmann A.A., Montgomery B.L., Popovici J., Iturbeormaetxe I., Johnson P.H., Muzzi F., Greenfield M., Durkan M., Leong Y.S., Dong Y. (2011). Successful establishment of *Wolbachia* in *Aedes* populations to suppress dengue transmission. Nature.

[21] O’Neill S.L., Ryan P.A., Turley A.P., Wilson G., Retzki K., Iturbe-Ormaetxe I., Dong Y., Kenny N., Paton C.J., Ritchie S.A. (2019). Scaled deployment of *Wolbachia* to protect the community from dengue and other *Aedes* transmitted arboviruses. Gates Open Res..

[22] Marks E.N. (1966). An Atlas of Common Queensland Mosquitoes; with a Guide to Common Queensland Biting Midges by E. J. Reye.

[23] Peterson B.K., Weber J.N., Kay E.H., Fisher H.S., Hoekstra H.E. (2012). Double digest RADseq: An inexpensive method for de novo SNP discovery and genotyping in model and non-model species. PLoS ONE.

[24] Korneliussen T.S., Albrechtsen A., Nielsen R. (2014). ANGSD: Analysis of Next Generation Sequencing Data. BMC Bioinform..

[25] Malinsky M., Trucchi E., Lawson D.J., Falush D. (2018). RADpainter and fineRADstructure: Population Inference from RADseq Data. Mol. Biol. Evol..

[26] Catchen J., Hohenlohe P.A., Bassham S., Amores A., Cresko W.A. (2013). Stacks: An analysis tool set for population genomics. Mol. Ecol..

[27] Pickrell J.K., Pritchard J.K. (2012). Inference of population splits and mixtures from genome-wide allele frequency data. PLoS Genet..

[28] Aubry F., Dabo S., Manet C., Filipović I., Rose N.H., Miot E.F., Martynow D., Baidaliuk A., Merkling S.H., Dickson L.B. (2020). Enhanced Zika virus susceptibility of globally invasive *Aedes aegypti* populations. Science.

[29] Jombart T., Devillard S., Balloux F. (2010). Discriminant analysis of principal components: A new method for the analysis of genetically structured populations. BMC Genet..

[30] Jombart T. (2008). adegenet: A R package for the multivariate analysis of genetic markers. Bioinformatics.

[31] Weir B.S., Goudet J. (2017). A Unified Characterization of Population Structure and Relatedness. Genetics.

[32] Rougemont Q., Moore J.-S., Leroy T., Normandeau E., Rondeau E.B., Withler R.E., Van Doornik D.M., Crane P.A., Naish K.A., Garza J.C. (2020). Demographic history shaped geographical patterns of deleterious mutation load in a broadly distributed Pacific Salmon. PLoS Genet..

[33] Kitada S., Nakamichi R., Kishino H. (2021). Understanding population structure in an evolutionary context: Population-specific *F*_ST_ and pairwise *F*_ST_. G3 GenesGenomesGenetics.

[34] Do C., Waples R.S., Peel D., Macbeth G.M., Tillett B.J., Ovenden J.R. (2014). NeEstimator v2: Re-implementation of software for the estimation of contemporary effective population size (*N_e_*) from genetic data. Mol. Ecol. Resour..

[35] McMillan L.F., Fewster R.M. (2017). Visualizations for genetic assignment analyses using the saddlepoint approximation method. Biometrics.

[36] Mordecai E.A., Caldwell J.M., Grossman M.K., Lippi C.A., Johnson L.R., Neira M., Rohr J.R., Ryan S.J., Savage V., Shocket M.S. (2019). Thermal biology of mosquito-borne disease. Ecol. Lett..

[37] White M.T., Griffin J.T., Churcher T.S., Ferguson N.M., Basáñez M.-G., Ghani A.C. (2011). Modelling the impact of vector control interventions on Anopheles gambiae population dynamics. Parasites Vectors.

[38] Roth I.H. (2012). The Discovery and Settlement of Port Mackay, Queensland.

[39] Filipović I., Hapuarachchi H.C., Tien W.-P., Razak M.A.B.A., Lee C., Tan C.H., Devine G.J., Rašić G. (2020). Using spatial genetics to quantify mosquito dispersal for control programs. BMC Biol..

[40] Guerra C.A., Reiner R.C., Perkins T.A., Lindsay S.W., Midega J.T., Brady O.J., Barker C.M., Reisen W.K., Harrington L.C., Takken W. (2014). A global assembly of adult female mosquito mark-release-recapture data to inform the control of mosquito-borne pathogens. Parasites Vectors.

[41] Trewin B.J., Pagendam D.E., Zalucki M.P., Darbro J.M., Devine G.J., Jansen C.C., Schellhorn N.A. (2020). Urban Landscape Features Influence the Movement and Distribution of the Australian Container-Inhabiting Mosquito Vectors *Aedes aegypti* (Diptera: Culicidae) and *Aedes notoscriptus* (Diptera: Culicidae). J. Med. Entomol..

[42] Whelan P.I., Kurucz N., Pettit W.J., Krause V. (2020). Elimination of *Aedes aegypti* in northern Australia, 2004–2006. J. Vector Ecol..

[43] Gubler D.J. (1998). Dengue and dengue hemorrhagic fever. Clin. Microbiol. Rev..

[44] Lee Y., Schmidt H., Collier T.C., Conner W.R., Hanemaaijer M.J., Slatkin M., Marshall J.M., Chiu J.C., Smartt C.T., Lanzaro G.C. (2019). Genome-wide divergence among invasive populations of Aedes aegypti in California. BMC Genom..

[45] Marshall J.M., Yang S., Bennett J.B., Filipović I., Rašić G. (2025). Spatial close-kin mark-recapture methods to estimate dispersal parameters and barrier strength for mosquitoes. PLoS Comput. Biol..

[46] Rašić G., Marshall J.M. (2026). Integrating mosquito genomics into simulation modeling: Opportunities for better-informed biocontrol. Curr. Opin. Insect Sci..

[47] Williams C.R., Bader C.A., Kearney M.R., Ritchie S.A., Russell R.C. (2010). The extinction of dengue through natural vulnerability of its vectors. PLoS Negl. Trop. Dis..

[48] Pagendam D.E., Trewin B.J., Snoad N., Ritchie S.A., Hoffmann A.A., Staunton K.M., Paton C., Beebe N. (2020). Modelling the Wolbachia incompatible insect technique: Strategies for effective mosquito population elimination. BMC Biol..

[49] Bansal S., Lim J.T., Chong C.-S., Dickens B., Ng Y., Deng L., Lee C., Tan L.Y., Kakani E.G., Yoong Y. (2024). Effectiveness of Wolbachia-mediated sterility coupled with sterile insect technique to suppress adult *Aedes aegypti* populations in Singapore: A synthetic control study. Lancet Planet. Health.

[50] Turley A.P., Zalucki M.P., O’nEill S.L., McGraw E.A. (2013). Transinfected *Wolbachia* have minimal effects on male reproductive success in *Aedes aegypti*. Parasites Vectors.

[51] Axford J.K., Ross P.A., Yeap H.L., Callahan A.G., Hoffmann A.A. (2016). Fitness of *w*AlbB *Wolbachia* Infection in *Aedes aegypti*: Parameter Estimates in an Outcrossed Background and Potential for Population Invasion. Am. J. Trop. Med. Hyg..

[52] Lau M.-J., Ross P.A., Hoffmann A.A. (2021). Infertility and fecundity loss of *Wolbachia*-infected *Aedes aegypti* hatched from quiescent eggs is expected to alter invasion dynamics. PLoS Negl. Trop. Dis..

[53] Yeap H.L., Mee P., Walker T.W., Weeks A.R., Oneill S.L., Johnson P.R.S., Ritchie S.A., Richardson K.M., Doig C.J., Endersby N.M. (2011). Dynamics of the “popcorn” *Wolbachia* infection in outbred *Aedes aegypti* informs prospects for mosquito vector control. Genetics.

[54] Nguyen T.H., Le Nguyen H., Nguyen T.Y., Vu S.N., Tran N.D., Le T.N., Vien Q.M., Bui T.C., Le H.T., Kutcher S. (2015). Field evaluation of the establishment potential of wMelPop *Wolbachia* in Australia and Vietnam for dengue control. Parasites Vectors.

[55] Rašić G., Endersby N.M., Williams C., Hoffmann A.A. (2014). Using *Wolbachia*-based release for suppression of *Aedes* mosquitoes: Insights from genetic data and population simulations. Ecol. Appl..

[56] Lim J.T., Mailepessov D., Chong C.-S., Dickens B., Lai Y.L., Ng Y., Deng L., Lee C., Tan L.Y., Chain G. (2024). Assessing *Wolbachia*-mediated sterility for dengue control: Emulation of a cluster-randomized target trial in Singapore. J. Travel Med..

[57] Lim J.T., Chong C.-S., Chang C.-C., Mailepessov D., Dickens B., Lai Y.L., Deng L., Lee C., Tan L.Y., Chain G. (2026). Dengue Suppression by Male *Wolbachia*-Infected Mosquitoes. N. Engl. J. Med..

[58] Zeng Q., She L., Yuan H., Luo Y., Wang R., Mao W., Wang W., She Y., Wang C., Shi M. (2022). A standalone incompatible insect technique enables mosquito suppression in the urban subtropics. Commun. Biol..

[59] Kiware S.S., Chitnis N., Tatarsky A., Wu S., Castellanos H.M.S., Gosling R., Smith D., Marshall J.M. (2017). Attacking the mosquito on multiple fronts: Insights from the Vector Control Optimization Model (VCOM) for malaria elimination. PLoS ONE.

[60] Vásquez V.N., Kueppers L.M., Rašić G., Marshall J.M. (2023). wMel replacement of dengue-competent mosquitoes is robust to near-term climate change. Nat. Clim. Change.

[61] Metzger M.E., Hardstone Yoshimizu M., Padgett K.A., Hu R., Kramer V.L. (2017). Detection and Establishment of *Aedes aegypti* and *Aedes albopictus* (Diptera: Culicidae) Mosquitoes in California, 2011–2015. J. Med. Entomol..

[62] Mann M.E., Gleick P.H. (2015). Climate change and California drought in the 21st century. Proc. Natl. Acad. Sci. USA.

[63] (2012). Assembly Bill No. 1750, Solorio, Chapter 537, Division 6 of the Water Code, Rainwater Capture Act of 2012. California Legislative Information. https://leginfo.legislature.ca.gov/faces/billTextClient.xhtml?bill_id=201120120AB1750.

[64] Johnson P.H., Spitzauer V., Ritchie S.A. (2012). Field Sampling rate of BG-sentinel traps for *Aedes aegypti* (Diptera: Culicidae) in suburban Cairns, Australia. J. Med. Entomol..

[65] Otero M., Solari H.G., Schweigmann N. (2006). A stochastic population dynamics model for *Aedes aegypti*: Formulation and application to a city with temperate climate. Bull. Math. Biol..

[66] Simoy M., Simoy M., Canziani G. (2015). The effect of temperature on the population dynamics of *Aedes aegypti*. Ecol. Model..

[67] Focks D.A., Haile D.G., Daniels E., Mount G.A. (1993). Dynamic life table model for *Aedes aegypti* (Diptera: Culicidae): Analysis of the literature and model development. J. Med. Entomol..

[68] Fay R.W. (1964). The biology and bionomics of *Aedes aegypti* in the laboratory. Mosq. News.

