# Supplementary material for: Eliminating Aedes aegypti from Its Southern Margin in Australia: Insights from Genomic Data and Simulation Modeling
Source: Insects. 2026 Jun 13;17(6):623. doi: 10.3390/insects17060623 (PMC13299893; doi:10.3390/insects17060623)
Supplement: Supplementary file 1 [file insects-17-00623-s001.zip › insects-4312828-supplementary.pdf]

Supplementary Materials for

Eliminating *Aedes aegypti* from Its Southern Margin in Australia: Insights from Genomic Data and Simulation Modeling

Gordana Rašić*, Igor Filipović, Sean L. Wu, Tomás M. León, Jared B. Bennett, Héctor M. Sánchez C, John M. Marshall, Brendan J. Trewin

*Correspondence: rasic.gordana@gmail.com or gordana.rasic@qimrb.edu.au

This PDF file includes:

Supplementary text.
Figures S1 to S2
Tables S1 to S2
Legends to data files S1 to S2
References

Other Supplementary Materials for this manuscript includes the following:

Data file S1
Data file S2

Files in this Data Supplement

Data_file_s1.txt
Data_file_s2.vcf

Supplementary Text

The average number of ~6 adult females per household assumed in the MGDrivE2 simulations was obtained from the entomological surveillance data in Wondai. Specifically, an average of 0.7 adult *Aedes aegypti* were caught per BG Sentinel trap per week in this town over a 9-week surveillance effort in 2018 (Fig. S1). Assuming that BGS traps have a 5-30% efficacy of capturing *Ae. aegypti* and that 2/3 of captured mosquitoes are females [64], the estimated total number of females per household is ~2-9.

Figure S1.

Mosquito numbers in Wondai, WBBR. The average number of *Aedes aegypti* per week per BG Sentinel trap during nine weeks in 2018.

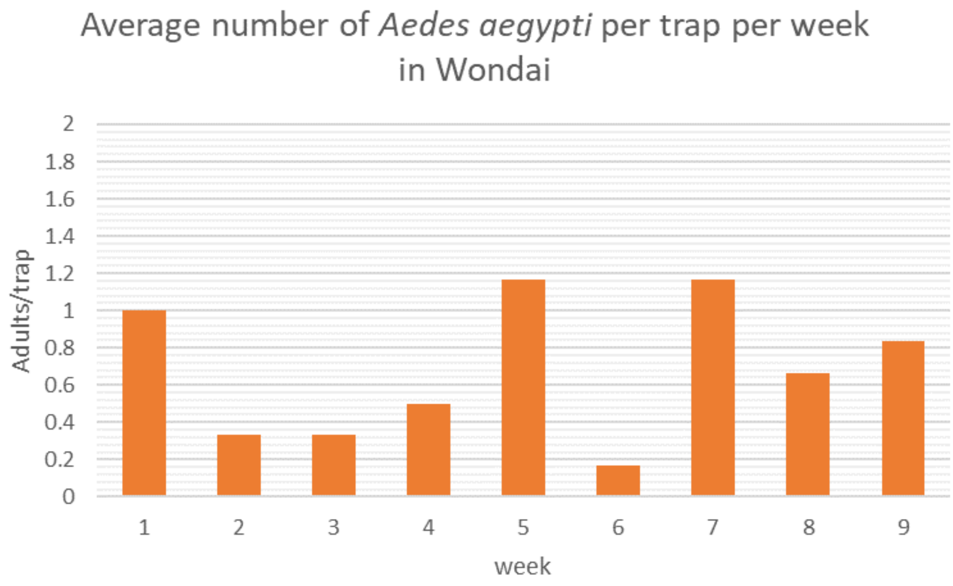

Figure S2. **IIT releases with different sex sorting error rates.** IIT releases without tank sealing are modeled in Murgon for overflooding ratios of 14:1, 20:1, 30:1 and 40:1, and the sex-sorting error of (A) 10^{-6}, (B) 10^{-7}, (C) 10^{-8}, (D) 10^{-9}. *Wolbachia* establishment is predicted only for IIT releases where the sex sorting error is $\geq 10^{-7}$ and overflooding ratio is high (>20:1).

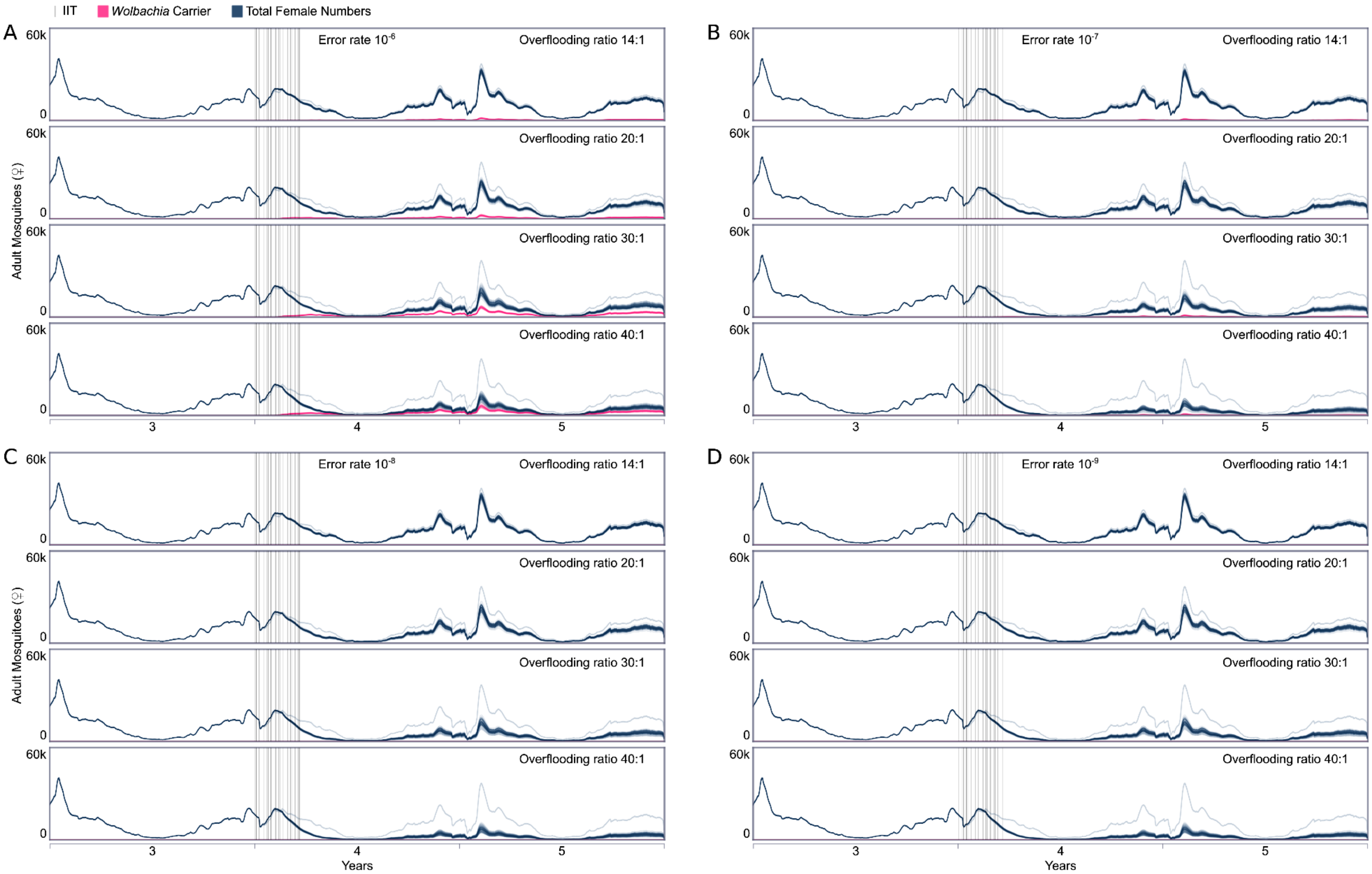

Figure S3.
Mosquito numbers before and after the tank-sealing campaign in Goomeri, WBBR. The total number of *Aedes aegypti* caught in BGSentinel traps during five weeks in 2018 prior to a tank-sealing campaign, and during the same period in the following year (2019). The observed reduction in mosquito numbers (20-25%) was used this to calibrate the simulations of larval carrying capacity in MGDrivE2.

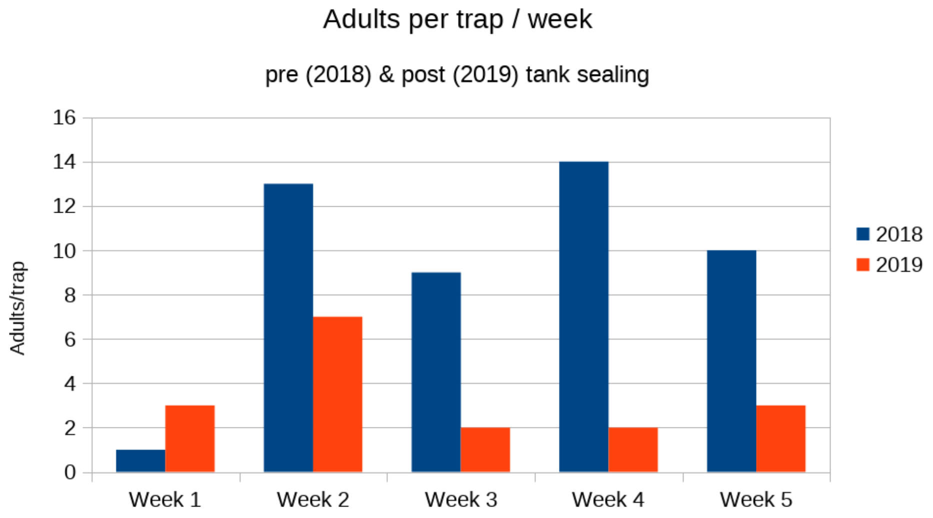

Table S1.
Entomological surveillance data. Results from the surveillance activities in the Wide Bay Burnett Region (WBBR) over 4-10 weeks in 2018-19, and the survey years since 2004 with *Aedes aegypti* presence/absence.

Town	% positive premises (n_p)	% positive ovitraps (n_o)	Weeks of trapping	Years present since 2004	Years absent since 2004
Wondai	40 (10)	20 (40)	10	2013,-17,-18	2004,-14
Murgon	29 (7)	57 (28)	10	2013,-17,-18	2006
Goomeri	57 (7)	32 (28)	9	2004,-14,-18	0
Gayndah	60 (5)	20 (20)	5	2004,-14,-18	0
Mundubbera	100 (5)	100 (24)	4	2011,-12,-18	0
Biggenden	60 (5)	100 (23)	6	2011,-12,-18	0
Gin Gin	0 (8)	0 (32)	10	2011-17	2004,-18,-19
Monto	100 (5)	100 (36)	6	2011,-12,-18	0

Table S2.

Average mosquito life cycle parameters. Time-varying parameters were scaled [65-68] such that their mean values over the simulation period correspond to the values in this table.

Parameter	Value	Reference
Egg Duration (days)	5	(2)
Larval Duration (days)	6	(2)
Pupal Duration (days)	4	(2)
Egg production per female (day^{-1})	20	(3)
Daily population growth rate (day^{-1})	1.175	(4)
Daily adult mortality rate (day^{-1})	0.09	(5,6)

Data S1. (separate file)

Variant Call Format (VCF) file (**Data_file_s1.vcf**) with genotypes across >15,000 autosomal loci for 229 *Aedes aegypti* analyzed in this study.

Data S2. (separate file)

Tab-delimited table (**Data_file_s2.txt**) showing centroid coordinates for each residential block, the number of households per block and the number of human hosts per block in Murgon and Wondai. These data were used to calibrate the spatially-explicit simulations in MGDrivE 2.
